# Decoupling Lineage-Associated Genes in Acute Myeloid Leukemia Reveals Inflammatory and Metabolic Signatures Associated With Outcomes

**DOI:** 10.3389/fonc.2021.705627

**Published:** 2021-08-04

**Authors:** Hussein A. Abbas, Vakul Mohanty, Ruiping Wang, Yuefan Huang, Shaoheng Liang, Feng Wang, Jianhua Zhang, Yihua Qiu, Chenyue W. Hu, Amina A. Qutub, Monique Dail, Christopher R. Bolen, Naval Daver, Marina Konopleva, Andrew Futreal, Ken Chen, Linghua Wang, Steven M. Kornblau

**Affiliations:** ^1^Department of Leukemia, The University of Texas MD Anderson Cancer Center, Houston, TX, United States; ^2^Department of Bioinformatics and Computational Biology, The University of Texas MD Anderson Cancer Center, Houston, TX, United States; ^3^Department of Genomic Medicine, The University of Texas MD Anderson Cancer Center, Houston, TX, United States; ^4^Department of Biostatistics & Data Science, School of Public Health, University of Texas Health Science Center at Houston, Houston, TX, United States; ^5^Department of Computer Science, Rice University, Houston, TX, United States; ^6^Department of Bioengineering, Rice University, Houston, TX, United States; ^7^Oncology Biomarker Development, Genentech Inc, South San Francisco, CA, United States; ^8^Oncology Bioinformatics, Genentech Inc, South San Francisco, CA, United States

**Keywords:** acute myeloid leukemia, lineage, metabolism, inflammation, multiplatform analysis

## Abstract

Acute myeloid leukemia (AML) is a heterogeneous disease with variable responses to therapy. Cytogenetic and genomic features are used to classify AML patients into prognostic and treatment groups. However, these molecular characteristics harbor significant patient-to-patient variability and do not fully account for AML heterogeneity. RNA-based classifications have also been applied in AML as an alternative approach, but transcriptomic grouping is strongly associated with AML morphologic lineages. We used a training cohort of newly diagnosed AML patients and conducted unsupervised RNA-based classification after excluding lineage-associated genes. We identified three AML patient groups that have distinct biological pathways associated with outcomes. Enrichment of inflammatory pathways and downregulation of *HOX* pathways were associated with improved outcomes, and this was validated in 2 independent cohorts. We also identified a group of AML patients who harbored high metabolic and mTOR pathway activity, and this was associated with worse clinical outcomes. Using a comprehensive reverse phase protein array, we identified higher mTOR protein expression in the highly metabolic group. We also identified a positive correlation between degree of resistance to venetoclax and mTOR activation in myeloid and lymphoid cell lines. Our approach of integrating RNA, protein, and genomic data uncovered lineage-independent AML patient groups that share biologic mechanisms and can inform outcomes independent of commonly used clinical and demographic variables; these groups could be used to guide therapeutic strategies.

## Introduction

Acute myeloid leukemia (AML) is a clinically and morphologically heterogeneous disease with significant variability in treatment responses and outcomes (1–3). Although almost 60-70% of AML patients achieve remission with standard anthracycline (idarubicin or daunorubicin) and cytarabine-based induction chemotherapy, almost 50% of these patients eventually experience relapse within 1 year of diagnosis (2–4). Revealing the underlying biologic processes that contribute to AML heterogeneity and drive outcomes may guide therapeutic strategies.

The French American British (FAB) classification was traditionally used to categorize AML into 8 different morphologic subtypes (M0 to M7) that reflected lineage commitment (2, 5–7). With the advent of cytogenetic and genomic assessments, the European Leukemia Network (ELN) recommendations were widely adopted as it proposed a risk stratification for patients that based on cytogenetics and genomics (2, 8, 9). However, cytogenetic and molecular alterations do not fully account for the heterogeneity of AML because not all patients harbor ELN–pre-defined aberrations (2, 10, 11). Also, there is considerable patient-to-patient variability in response to treatment and clinical outcomes within genomic and ELN subgroups (11). Therefore, there is a need to uncover underlying biologic pathways that are underrepresented in genomic and cytogenetic profiling of AML and may inform outcomes.

To fill this gap, researchers have identified several transcriptomic signatures associated with AML clinical outcomes (10, 12–17). However, RNA-based profiling revealed that this method of grouping AML patients was highly associated with FAB classifications, i.e., related to AML morphology and lineages (15, 16, 18). Yet, the FAB-associated clustering was not accounted for in previous transcriptome-based studies, suggesting that the morphology and lineage of AML were driving patient grouping. Furthermore, although mutations were associated with some transcriptomic-based clustering, there was significant overlap for these mutations in multiple clusters (15, 16). We hypothesized that by decoupling the lineage-related genes from the transcriptomic profiles of AML, we could unmask biologically relevant pathways that are inherent to AML independent of cell of origin and that could inform clinical outcomes. Furthermore, such an approach could identify biologic pathways associated with cluster-specific mutations.

In the current study, we decoupled FAB-associated genes to decipher lineage-independent biologic pathways in 81 newly diagnosed and previously untreated AML patients. We identified distinct biologic AML patient groups and assessed the outcome of patients according to their group membership. To provide further molecular orthogonal characterization of defined groups, we applied a reverse phase protein array (RPPA) in all patient samples and extended panel DNA sequencing in 73 of 81 patients (90%). Using this approach, we identified inflammatory and metabolic pathways associated with outcomes and validated our findings in 2 independent AML cohorts. The findings from this work demonstrate that RNA-based classification could reveal important potentially targetable biologic pathways.

## Methods

### Patient Population

A total of 81 newly diagnosed AML patients evaluated at The University of Texas MD Anderson Cancer Center were included in the current study. All patients had bone marrow samples collected and analyzed prior to treatment initiation. Patients provided written informed consent that was approved by the MD Anderson Institutional Review Board. The study was conducted in accordance with the Declaration of Helsinki.

### RNA Sequencing and Processing

RNA was isolated and purified from bone marrow mononuclear cells using Qiagen’s RNAEasy preparation kits. The purified RNA was used to create cDNA libraries that were assayed using TruSeq (Illumina) RNAAccess. For each sample, 40M 50-bp paired-end reads were sequenced using the Illumina HiSeq sequencer. RNA sequencing (RNA-seq) FASTQ files were processed through FastQC (v0.11.5) and RNA-SeQC (v1.1.8) (1) to generate a series of RNA-seq–related quality control metrics. STAR 2-pass alignment (v2.5.3) (2) was performed with default parameters to generate RNA-seq BAM files. Normalized counts were generated using DESEq2, then log_2_-transformed.

### Differential Expression and Pathway Analysis

In our cohort and TCGA cohort, gene-level read counts were used to perform differential expression analysis using *DESeq2 *(3). The T-statistic from the differential expression analysis was used to perform gene set enrichment analysis (GSEA) using the Bioconductor package *gage *(4), and significantly dysregulated pathways were identified at q < 0.1. For the Valk et al. validation cohort, we used single-sample GSEA (ssGSEA) (5, 6) implemented in the Bioconductor package *GSVA* (7) to convert microarray gene expression into pathway activity scores. Pathway activity scores were compared between groups using the Wilcox Rank Sum test, and p-values were corrected for multiple hypothesis testing using false discovery rate (FDR). Significantly dysregulated pathways were identified at q < 0.05. The Hallmark pathways (8) were used in GSEA and ssGSEA.

### Unsupervised Clustering Prior to Excluding FAB-Associated Genes

The R package *pheatmap* was used to generate heatmaps and dendrograms for samples from a matrix of variably expressed genes. Euclidian distance and complete clustering were used to perform hierarchical clustering of the data. We used a dynamic tree cutting algorithm implemented in the *cutreeDynamic*() function from the *WGCNA* package (9) to identify optimal clustering of patients. The strength of association between cluster membership and FAB status (M1/M2 and M4/M5) was tested using Fisher’s test. Clustering analysis in our dataset was performed using an increasing number of top variably expressed genes (1000, 1500, 2000, 2500, and 3000 genes). Clustering analysis in TCGA cohort was performed as above using the top 1000 variably expressed genes, including only samples with corresponding FAB status (M1/M2 and M4/M5).

### Unsupervised Clustering to Identify Patient Clusters Independent of FAB Status

**Figure 1:**
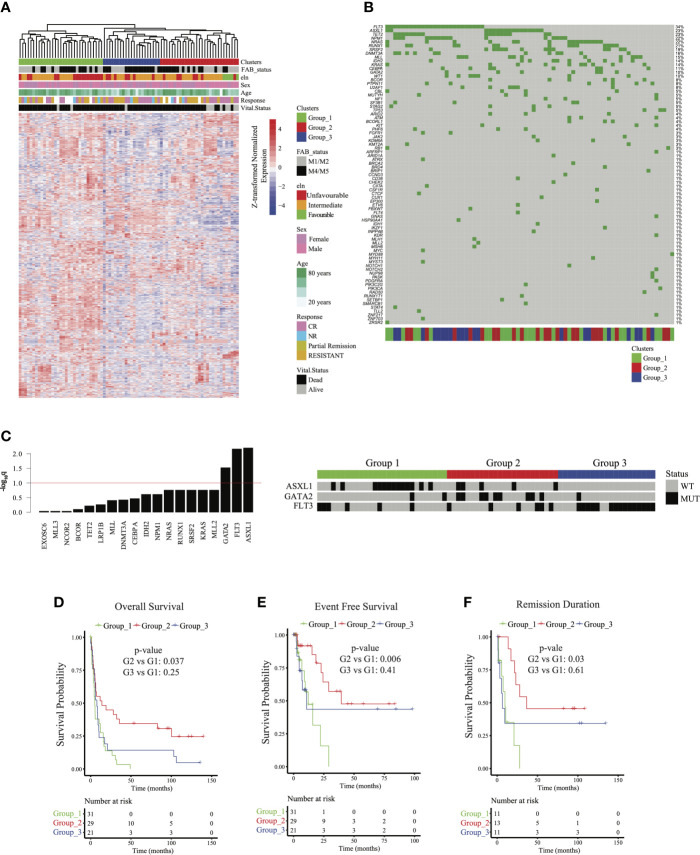
Identifying acute myeloid leukemia patient groups independent of French American British (FAB) classification: **(A)** Clustered gene expression heatmap of top variably expressed genes (variance > 5,735 genes) whose expression was not associated with FAB classification (Fisher p = 0.251). ELN = European Leukemia Network; CR = complete remission. **(B)** Oncoplot of frequently mutated genes in the cohort. **(C)** (**left**) Barplot of –log_10_ Fisher test q values testing the association of mutations with sample groups. *ASXL1*, *GATA2*, and *FLT3* mutations were associated with groups 1, 2, and 3, respectively (q < 0.1). (**right**) Heatmap showing mutation status of *ASXL1*, *GATA2*, and *FLT3* among patients. WT = wild type; MUT = mutated. **(D)** Overall survival, **(E)** event-free survival, and **(F)** remission duration of patients in groups 1, 2, and 3. p values for **(D–F)** were calculated using a multivariable Cox regression model relative to cluster 1.

To identify genes with expression associated with FAB status, we excluded genes associated with FAB and lineage commitment. Specifically, for each gene, a p-value was obtained for each of the FAB groups M2, M4, and M5 relative to M1 from the regression analysis. p-values for each of the groups (M2, M4, and M5) were corrected for multiple testing using FDR. A gene’s expression was considered associated with FAB status if expression of the gene was significantly different in at least one of the FAB groups M2, M4, and M5 relative to M1 (q < 0.05). A total of 4743 genes were found to have expression associated with FAB status. Enrichment of these genes in cell type-specific signatures was quantified using *Enricher (* 10, 11). FAB-associated genes were excluded, and the top variably expressed genes (variance > 5, 735 genes) were used to identify clusters (as described above). Three distinct expression clusters were identified using this approach, Group_1, Group_2 and Group_3 (**Figure 1A**)

### Survival Analysis

Survival analysis of patient clusters identified from our expression data (**Figures 1D–F**) was performed using multivariant Cox regression implemented in the R package *survival*. First, clinical variables important for survival were identified using univariate Cox regression. The multivariate survival model was built using all variables that were significantly associated with survival in the univariate analysis (p < 0.05). Survival analysis reported in **Figures 3A–C** and **Supplementary Figure 4** was performed using the *survminer R* package. p-values for the KM-plots were computed using log-rank test implemented in the function surv_pvalue(). All KM-plots in the study were plotted using the function ggsurplot().

### HOX Gene Survival Analysis

Activity of *HOXA* and *HOXB* gene clusters were scored in all datasets using ssGSEA (5, 6) implemented in *GSVA* (7). In each cohort, for each *HOX* gene cluster, the samples were split into 2 groups (the top and bottom 50^th^ percentile) based on the activity scores obtained from ssGSEA. Survival differences between the groups were quantified as described above.

### Estimation and Comparison of Metabolic Activity Between Patient Groups

Pathway activity scores were calculated using 91 gene sets, including 85 KEGG (12) metabolic pathways and 5 literature-curated gene sets: glucose deprivation, glycolysis, hypoxia, mTOR, and oxidative phosphorylation. The pathway activity score was calculated using ssGSEA using GSVA (7). Differential activity of pathways among clusters was identified using the Wilcoxon rank-sum test based on each cell’s pathway activity scores. p-values were adjusted using the Benjamini-Hochberg method, and the threshold of significance was set to q < 0.05.

### Foundation Medicine Assay

Samples were submitted to a Clinical Laboratory Improvement Amendments–certified, New York State-accredited, and College of American Pathologists–accredited laboratory (Foundation Medicine, Cambridge, MA) for next-generation sequencing–based genomic profiling. Samples were processed in the protocol defined by hematologic cancers as previously described (13). Briefly, after DNA and RNA extraction from bone marrow aspirate, adaptor-ligated DNA underwent hybrid capture for all coding exons of 465 cancer-related genes. cDNA libraries prepared from RNA underwent hybrid capture for 265 genes known to be rearranged in cancer. Captured libraries were sequenced to a median exon coverage depth of >500× (DNA) or ~3M unique reads (RNA) using Illumina sequencing, and resultant sequences were analyzed for base substitutions, small insertions and deletions (indels), copy number alterations (focal amplifications and homozygous deletions), and gene fusions/rearrangements, as previously described (13, 14). Frequent germline variants from the 1000 Genomes Project (dbSNP142) were removed. To maximize mutation-detection accuracy (sensitivity and specificity) in impure clinical specimens, the test was previously optimized and validated to detect base substitutions at ≥5% mutant allele frequency, indels at ≥10% mutant allele frequency with ≥99% accuracy, and fusions occurring within baited introns/exons with >99% sensitivity (14). Known confirmed somatic alterations deposited in the Catalog of Somatic Mutations in Cancer (COSMIC v62) were called at allele frequencies ≥1% (15). Patients did not provide consent for raw data release. Therefore, associated raw sequence data is not shared. However, variants from a subset of the samples used in this analysis (>18,000) have been deposited in the Genomic Data Commons (accession #phs001179).

### Mutational Analysis

Mutation data were binarized to indicate the presence or absence of a mutation. Genes mutated in less than 10% of the samples were excluded from the analysis. Fisher’s test was used to quantify the association between the presence of a gene mutation and cluster membership. p-values were corrected using FDR and mutations significantly associated with a cluster were identified at q < 0.1. Oncoplot was generated using R package *maftools* (16). Gene Mutations that showed significant association with FLT3 mutation were identified using Fisher’s test, p-values were corrected for multiple testing using FDR and significant associations were identified at q < 0.1. An odds ratio (OR) > 1 indicates co-occurrence and OR < 1 indicates mutual exclusivity.

### Validation Cohorts

Data for the 2 validation datasets TCGA (n= 173) and Valk et al. (n= 293) (17, 18) were downloaded from GEO database (GSE1159) and UCSC Xena (https://xenabrowser.net/datapages/). Clinical data were also available on GEO for the Valk et al. cohort, and from Firehose (https://gdac.broadinstitute.org/) for The Cancer Genome Atlas (TCGA) cohort. To validate survival and pathway patterns observed in Group 2 we performed differential expression analysis between group 2 and groups 1 and 3 combined. Upregulated and downregulated genes were identified as fold-change >2 and <-2, respectively, at q < 0.05. The activity of these gene sets was scored in each of the validation cohorts using ssGSEA, and each sample was then assigned a score indicating the difference between the activity of the upregulated and downregulated gene sets. In each of the validation cohorts, samples were stratified into 2 groups indicating more Group 2 like (top 50^th^ percentile) and less Group 2 like (bottom 50^th^ percentile). These 2 groups were then used to perform survival analysis as described above. Differential expression between the groups was determined and pathway analysis performed as described above.

### Differential Expression of Proteins

RPPA data used in the study were previously published and generated by our group for the cohort of 81 patients (19). To identify differentially expressed proteins between 2 groups, we computed the difference in mean expression of each protein with a p-value using the Wilcoxon rank-sum test. p-values were corrected using FDR. Upregulated and downregulated proteins were identified as difference in mean >75th percentile and <25th percentile, respectively, at q < 0.1.

### Cell Line Molecular and Drug Response Data

Cancer cell line drug response data were obtained from Rees et al. (20). The response of each cell to a drug was quantified as the area under the drug response curve (AUC). High AUC indicated poor response and low AUC indicated better response. Protein expression from RPPA for these cell lines was obtained from Cancer Cell Line Encyclopedia (21) using the DepMap portal (https://depmap.org/portal/download/). Correlations between expression data and drug response were computed using Spearman correlation.

## Results

### Clinical and Demographic Characteristics

A total of 81 newly diagnosed AML patients (58% male and 42% female) with a median age of 67.0 years (range 17.4-85.2 years) were included in the study. All patients had whole transcriptome sequencing and RPPA profiling at the time of diagnosis, and 73 of 81 patients had targeted sequencing of 465 genes using Foundation Medicine’s FoundationOne Heme assay. Patient clinical and demographic characteristics are summarized in **Table 1**. Briefly, 46 patients (57%) had intermediate cytogenetic risk per ELN risk assessment (22), 30 (37%) had unfavorable risk, and 5 (6%) had favorable risk. A total of 36 patients (44%) were classified as M1/M2 and 45 patients (56%) were classified as M4/M5 by FAB classification. Thirty-three patients (41%) had antecedent hematologic disorder. Eleven patients were alive at the time of this analysis, with a median follow-up period of 388.1 weeks (range 0-559.5 weeks). Eighty percent of patients (56/70 for whom data were available) were treated with cytarabine-based regimens, 13% were treated with hypomethylating agents (9/70), and 7% with investigational treatments (5/70). Eleven patients had no treatment records at MD Anderson. Of those evaluable for response, 35/63 (56%) had complete remission or a partial response (complete remission: 33/35, 94%; partial response: 2/35, 6%), and 28/63 (44%) had primary refractory disease. Among the patients who had complete remission or a partial response, 20/35 patients (57%) eventually had a relapse. The median overall survival, event free survival, and remission duration for all evaluable patients were 25.4 weeks (range 0-559.6 weeks), 22.4 weeks (range 0-393 weeks), and 42.4 weeks (range 3.3-538.7 weeks), respectively (**Supplementary Figures 1A–C**).

**Table 1:** Clinical and demographic characteristics of patients.

Characteristic	No. (%)							
	Overall, n = 81	Group 1, n = 31		Group 2, n = 29		Group 3, n = 21	p*^1^*	q*^2^*
Mean ± SD age, years	64.3±14.1	68.9±9.9	64.4±15.1		57.3±15.6		0.030	0.073
Sex							0.250	0.370
Female	34/81 (42)	12/31 (39)	10/29 (34)		12/21 (57)			
Male	47/81 (58)	19/31 (61)	19/29 (66)		9/21 (43)			
FAB							0.230	0.370
M1/M2	36/81 (44)	16/31 (52)	14/29 (48)		6/21 (29)			
M4/M5	45/81 (56)	15/31 (48)	15/29 (52)		15/21 (71)			
ELN genetic group							0.019	0.073
Favorable	5/81 (6)	0/31 (0)	5/29 (17)		0/21 (0)			
Intermediate	46/81 (57)	15/31 (48)	17/29 (59)		14/21 (67)			
Unfavorable	30/81 (37)	16/31 (52)	7/29 (24)		7/21 (33)			
Recent AHD							0.180	0.360
No	48/81 (59)	16/31 (52)	16/29 (55)		16/21 (76)			
Yes	33/81 (41)	15/31 (48)	13/29 (45)		5/21 (24)			
Treatment							0.280	0.370
AraC-based	56/70 (80)	17/24 (71)	21/25 (84)		18/21 (86)			
HMA-based	9/70 (13)	4/24 (17)	4/25 (16)		1/21 (5)			
Investigational	5/70 (7)	3/24 (13)	0/25 (0)		2/21 (10)			
(Missing)	11	7	4		0			
Response							>0.99	>0.99
CR	33/70 (47)	10/24 (42)	12/25 (48)		11/21 (52)			
Not Evaluable	7/70 (10)	3/24 (13)	2/25 (8)		2/21 (10)			
Partial remission	2/70 (3)	1/24 (4)	1/25 (4)		0/21 (0)			
Resistant	28/70 (40)	10/24 (42)	10/25 (40)		8/21 (38)			
(Missing)	11	7	4		0			
Relapse	20/35 (57)	8/11 (73)	6/13 (46)		6/11 (55)		0.480	0.530
(Missing)	46	20	16		10			
Vital status							0.028	0.073
Alive	11/81 (14)	2/31 (6)	8/29 (28)		1/21 (5)			
Dead	70/81 (86)	29/31 (94)	21/29 (72)		20/21 (95)			
AlloSCT	7/81 (9)	1/31 (3)	4/29 (14)		2/21 (10)		0.370	0.440
Mean ± SD bone marrow blast percentage	60.0±23.1	55.2±22.9	51.9±22.2		79.1±12.2		<0.001	<0.001
(Missing)	1	0	0		1			

^1^Statistical tests performed: Kruskal-Wallis test; chi-square test of independence; Fisher exact test.

^2^False discovery rate correction for multiple testing.

FAB, French-American-British classification; ELN, European Leukemia Network; AHD, antecedent hematologic disorder; AraC, ara-cytarabine; HMA, hypomethylating agents; CR, complete remission; alloSCT, allogeneic stem cell transplantation.

### Unsupervised Clustering to Identify Prognostic Clusters Independent of FAB and ELN Classification

Unsupervised clustering of the 81 newly diagnosed AML patients based on the top 1000 variably expressed genes initially revealed two distinct patient clusters. The clustering was highly associated with FAB morphologic classification (Fisher p = 9.2e^-5^, **Supplementary Figure 2A**). The FAB-associated clustering of patients persisted even when more genes were added to the unsupervised clustering, suggesting a significant impact of lineage and morphology on transcriptomic-based clustering (**Supplementary Figure 2B**). To assess whether this observation was also relevant in other AML cohorts, we conducted unsupervised clustering of expression profiles in M1/M2 and M4/M5 patients from TCGA AML cohort (18). Indeed, unsupervised clustering of TCGA AML cohort revealed similar high dependency on FAB classification (Fisher p = 2.24e^-10^, **Supplementary Figure 2C**). These findings suggested that the genes associated with lineage morphology in AML were contributing to AML transcriptomic-based clustering, and hence could be masking AML subgroups that share similar underlying biology but different lineages.

To address this concern, we used linear regression models and identified genes whose expression profiles were associated with FAB classification (q < 0.05; see *Methods*). Using enrichment analysis in cell lineage and morphology signatures (10, 11), we found that these genes were highly enriched for myeloid and monocytic lineage differentiation (**Supplementary Figure 3A**). To decouple lineage-associated genes from AML patient clustering and to identify biologically similar AML patients independent of lineage, we excluded the lineage-associated genes and re-clustered AML patients based on the expression of top variable genes (735 genes, variance > 5). This approach led us to identify 3 distinct patient clusters (hereafter referred to as group 1, group 2, and group 3) that clustered independently of FAB classification (Fisher p = 0.251; **Figure 1A** and **Supplementary Figure 2B**). The clinical and demographic characteristics of these 3 clusters are summarized in **Table 1**. Briefly, after correction for multiple hypothesis testing, there were no significant differences in distribution for FAB classification, ELN classification, sex, antecedent hematologic disease, or treatment group. Group 1 patients were the oldest (mean age 68.9 ± 9.9 years), followed by group 2 patients (64.4 ± 15.1 years) and group 3 patients (57.3 ± 15.6 years; p = 0.030, q = 0.073). These findings suggested that the patient grouping was inherently driven by the transcriptomic signatures independent of lineage or clinical characteristics.

Targeted DNA sequencing of 465 genes using the FoundationOne Heme assay in 73 patients (90%) revealed *FLT3* (37%), *TET2* (30%), *ASXL1* (25%), *NPM1* (22%), and *NRAS* (22%) as the most commonly mutated genes in the cohort (**Figure 1B**). *ASXL1* (q = 0.006), *GATA2* (q = 0.029), and *FLT3* (q = 0.006) mutations were significantly enriched in groups 1, 2, and 3, respectively (**Figure 1C**), but these mutations were not associated with FAB classification (q > 0.4 for all). *FLT3 expression was highest in Group 3, but it was significantly different only when compared to Group 2 (q= 0.018)* (**Supplementary Figure 3B**). We found significant association between mutations in FLT3 and mutations in NPM1 (q = 0.094, OR: 3.82) and ASXL1 (q = 0.094, OR: 0.3) mutations (**Supplementary Figure 3C**). Of note, while a significant fraction of NPM1 mutations co-occur with FLT3, mutations in AXSL1 were largely mutually exclusive. We included this data as **Supplementary Figure 3B**. These findings suggested that mutation profiles were associated with transcriptomic signatures, but not with lineage.

### Outcomes of AML Patient Groups

We next evaluated whether the 3 AML groups had distinct clinical outcomes. Univariate Cox survival analysis indicated that sample clustering was associated with differential overall survival, event-free survival, and remission duration (p < 0.05). To control for other confounding clinical factors, we first used univariate survival analysis to identify clinical variables associated with survival (p < 0.05; **Supplementary Table 1**) and then built a multivariable Cox survival model with these variables. Survival trends observed in the clusters in univariate analysis were re-captured after controlling for other confounding variables associated with survival (see *Methods*). Group 2 was characterized by improved overall survival (median 55.86 weeks; p = 0.037), event-free survival (median 55.85 weeks; p = 0.006), and remission duration (median 111.71 weeks; p = 0.03 relative to group 1, whereas no significant difference was observed between group 1 and group 3 (**Figures 1D–F**).

### Inflammatory and Immune Pathways Enriched in Group 2 Patients

**Figure 2:**
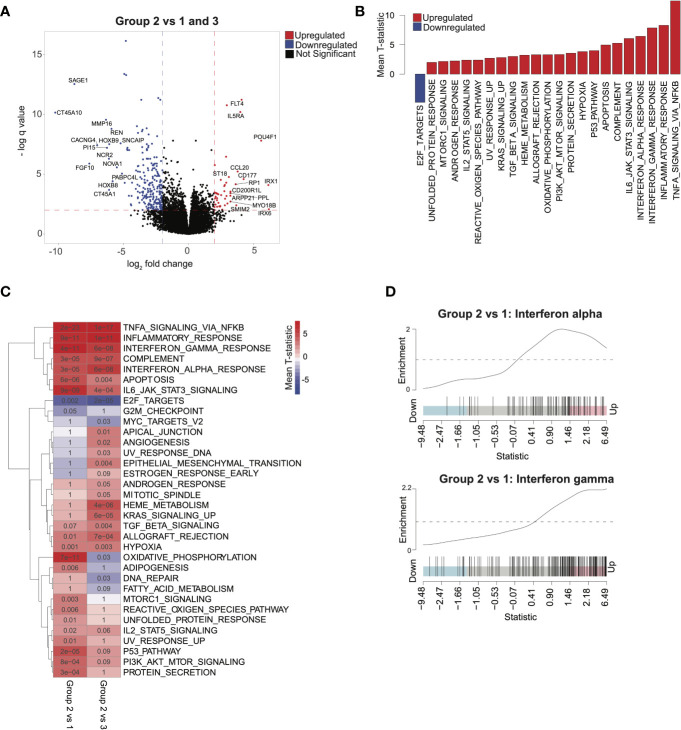
Characterizing transcriptomic features of acute myeloid leukemia patients in group 2: **(A)** Volcano plot corresponding to differential expression analysis comparing the transcriptome of group 2 with that of group 1 and 3 combined (significance based on log_2_ fold change > 2 and q < 0.05, in red). **(B)** Pathways identified *via* gene set enrichment analysis (GSEA) of significantly differentially expressed genes from **(A)**. Negative mean T-statistic (blue) indicates downregulation and positive mean T-statistic (red) indicates upregulation of the pathway. **(C)** GSEA mean T-statistic heatmap based on pairwise differential expression comparing group 2 with group 1 and with group 3. Pathways significantly dysregulated (q < 0.1) in at least one comparison are included in the heatmap. Red and blue indicate upregulation and downregulation, respectively. Numbers in the heatmap correspond to q values. **(D)** Barcode plots illustrating upregulation of interferon-alpha and interferon-gamma signaling in group 2 relative to group 1.

To explore transcriptomic signatures that were associated with improved outcomes in group 2, we conducted differential gene expression profiling comparing group 2 with groups 1 and 3, revealing 70 upregulated genes and 322 downregulated genes (q < 0.05, absolute log_2_ fold change > 2; **Figure 2A** and **Supplementary Table 1**). GSEA of hallmarks pathways demonstrated significant activation of immune signaling in group 2 compared with groups 1 and 3 (**Figure 2B**). To determine whether the signal was confounded by a single group, we next compared group 2 with group 1 and 3 each separately. Indeed, we saw that patients in group 2 consistently had activation of immune and inflammatory pathways, including interferon-alpha and interferon-gamma, compared with each of the other groups, suggesting that intrinsic immune activation in group 2 was associated with improved outcomes (**Figures 2C, D**). *HOXA* and *HOXB* gene clusters were significantly downregulated in group 2 compared with groups 1 and 3 (**Supplementary Table 1**). Furthermore, lower expression of *HOXA* and *HOXB* gene clusters was associated with better outcomes across all patients in our cohort (**Supplementary Figure 4A**).

### Validation of Immune Signatures in Independent Cohorts

**Figure 3:**
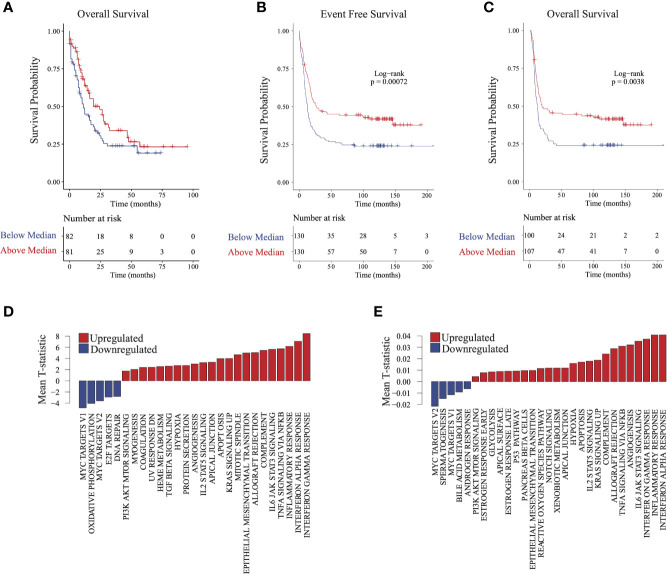
Validating survival and pathway trends observed in group 2 in independent cohorts: Samples in The Cancer Genome Atlas (TCGA) and Valk et al. cohorts were stratified on the basis of their similarity to group 2 (see *Methods*). **(A)** Overall survival and **(B)** event-free survival in TCGA cohort. **(C)** Overall survival in the Valk et al. cohort. **(D, E)** Gene set enrichment analysis barplots of TCGA **(D)** and Valk et al. **(E)** validation cohorts.

To validate the finding that immune signatures were associated with improved outcomes in AML, we used 2 independent AML cohorts (17, 18) with available transcriptomic and clinical data (**Supplementary Table 1**; see *Methods*). We then compared outcomes based on median scores derived from ssGSEA (7) from genes differentially expressed in group 2 relative to groups 1 and 3 (see *Methods*). Higher-scoring patients (i.e., more similar to group 2) had improved survival in both validation cohorts (**Figures 3A–C**). Differential pathway activity analysis between these groups revealed activation of immune-associated pathways, consistent with observations in group 2 in our cohort and further validating our finding that immune activity was the main differential factor in outcomes (**Figures 3D, E**). Similarly, patients with lower *HOXA* and *HOXB* gene scores had improved outcomes (**Supplementary Figures 4B, C**). These data indicate that activation of immune-associated pathways and suppression of *HOX* genes in AML are associated with improved outcomes in patients.

### Pairwise GSEA Comparisons Revealing Metabolic Signatures in Group 3

**Figure 4:**
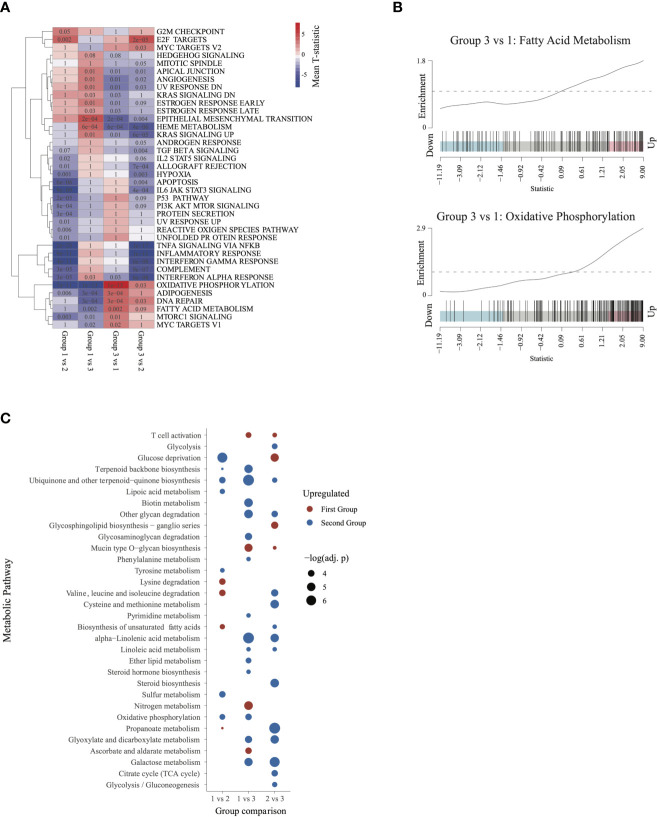
Characterizing the transcriptome of patients in group 3 and group 1: **(A)** Gene set enrichment analysis heatmap, similar to **Figure 2**. Pathways significantly dysregulated (q < 0.1) in at least one of the comparisons were included in the heatmap. **(B)** Barcode plots illustrating upregulation of fatty acid metabolism and oxidative phosphorylation in group 3 relative to group 1 patients. **(C)** Metabolic pathways differentially activated (q ≤ 0.05) in each of the 3 comparisons. Upregulated pathways are shown in red and downregulated pathways in blue (relative to the second group in each comparison).

To further characterize the biologic pathways that distinguished patient groups, we conducted pairwise GSEA between individual groups of patients. Group 3 patients had significant activation of metabolic activity compared with group 1 and with group 2 patients (**Figures 4A, B**). Although patients in group 3 and group 1 had similarly worse outcomes, activity of metabolic pathways was significantly higher in group 3 patients, especially for oxidative phosphorylation and fatty acid metabolism (**Figure 4B**), suggesting that metabolism was a distinguishing feature between these groups. Furthermore, activity in the mTOR pathway, a major regulator of cancer metabolism (23), was significantly higher in group 3 than in group 1.

To further characterize metabolic activity in group 3, we compared the metabolic pathway activity scores between group 3 and groups 2 and 1. Relative to group 2, group 3 showed significant activation of energy metabolism pathways such as glycolysis, TCA cycle, biosynthesis of unsaturated fatty acids, and gluconeogenesis. Relative to group 1, group 3 was characterized by activation of oxidative phosphorylation, lipid metabolism(ether lipid metabolism, steroid hormone biosynthesis), and pyrimidine metabolism. Group 3 also showed activation of galactose metabolism and linoleic acid metabolism relative to both group 1 and group 2. These findings indicate that patients in group 3 may be characterized by augmented activation of pathways involved in energy production and metabolism (**Figure 4C**).

### Proteomic Assessment to Distinguish Group 3 and Group 1

All 81 AML patients had previously reported RPPA profiling (19) at the same time point of RNA and genomic sequencing. We therefore used this orthogonal molecular platform to delineate protein-based molecular pathways that could differentiate these 3 AML patient groups (**Supplementary Figure 5**). Group 2 had downregulation (q = 0.057 and difference in mean = -0.832) of only CTNNB1 when compared with group 1 (**Supplementary Figure 5A**) and downregulation of MTOR and MTOR.pS2448 compared with group 3 (**Supplementary Figure 5B**). These findings suggested that unlike RNA, RPPA was not able to delineate many proteomic differences between group 2 and groups 1 and 3, most likely owing to the smaller number of genes assayed.

**Figure 5:**
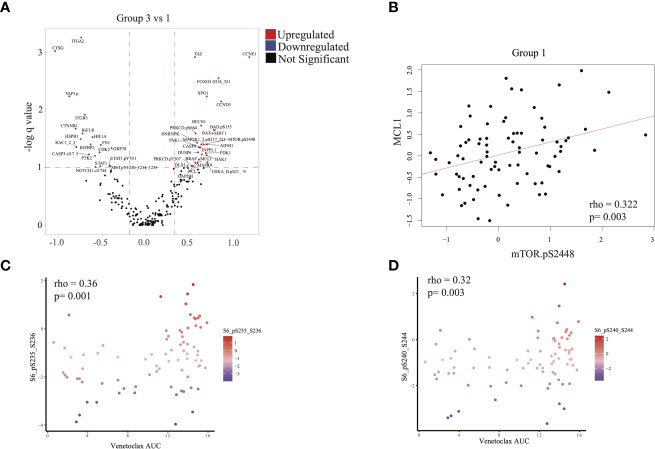
Proteomic analysis: **(A)** Differential protein expression analysis comparing group 3 with group 1. Upregulated proteins (q < 0.1 and difference in mean > 0.32) are shown in red and downregulated proteins (q < 0.1 and difference in mean > -0.188) in blue. **(B)** Scatterplot illustrating the correlation between expression of mTOR.pS2448 (activating phosphorylation) and MCL1 (spearman correlation = 0.322, p = 0.003). **(C, D)** Scatterplot illustrating the expression of phosphorylated S6 (marker of mTOR activation) with venetoclax (higher area under the curve [AUC] indicates more resistance to treatment). Statistics computed using Spearman correlation.

We next evaluated differences in RPPA signatures between group 1 and group 3 patients who had similar outcomes, compared with group 2 patients. We identified 28 upregulated proteins and 19 downregulated proteins (q < 0.1, see *Methods*) in group 3 relative to group 1 (**Figure 5A**). MTOR.pS2448, which signals for activation of both mTOR and PI3K pathways (23–25), was over-expressed in group 3. This was consistent with the mTOR upregulation in the group 3 transcriptomic signature and suggested an active PI3K-AKT-mTOR signaling axis in group 3 patients. In addition, we found over-expression of proteins in the MAPK signaling cascade (MAP2K1_2pS217_211, MAPK9), apoptosis (BAX, CASP8, MCL1, BAK1, BAD.pS155), and BRAF in group 3 compared with group 1 (**Figure 5A**). Overexpression of MCL1, accumulation of total (un-cleaved) CASP8, and lower expression of cleaved caspase-3 (CASP3.cI175) suggested inhibition of apoptosis in group 3 patients (**Figure 5A**), consistent with the higher absolute blast count observed in group 3 (**Table 1**).

MCL1 overexpression is associated with venetoclax resistance and can be seen in *FLT3*-mutated AML (26). We therefore checked for a correlation between mTOR and MCL1 expression. Indeed, MTOR.pS2448 expression was positively correlated with MCL1 expression in RPPA across all patients (**Figure 5B** and **Supplementary Figure 5B**). This is significant because resistance to venetoclax can be mediated *via* MCL1 (26). We therefore evaluated whether resistance to venetoclax in myeloid and lymphoid cell lines is also associated with mTOR overexpression. Phosphorylated S6 p235-236 and p240-244, which are surrogate markers for mTOR activation, were positively correlated with venetoclax AUC (ρ = 0.36, p = 0.001 and ρ = 0.32, p = 0.003, respectively), suggesting that mTOR activation was associated with resistance to venetoclax (**Figures 5C, D**).

## Discussion

Clinical outcomes of AML patients are largely determined by patient characteristics such as age, performance status, and the underlying cause of the AML (27). ELN classification categorizes AML patients on the basis of cytogenetic and mutational profiles (22, 28). However, almost one-third of AML patients lack prognostic genomic features (29). Also, one-third of AML patients have survival outcomes that deviate more than 20% from their ELN risk category (29). Therefore, identifying orthogonal molecular approaches contributing to AML heterogeneity independent of clinical and genomic features may reveal biologic processes impacting outcomes and identify novel therapeutic strategies.

In previous studies using RNA profiling to classify AML patients (17, 30), gene expression clustering was strongly correlated with mutational and cytogenetic profiles, as well as lineage and morphologic groups as classified by FAB. Similarly, we identified cluster correlation with FAB classification in TCGA and in our cohort. We therefore hypothesized that by excluding the expression profiles of genes associated with lineage and morphologic characteristics in AML, we can potentially uncover AML patient groups that share biologic pathways independent of morphology and lineage. In the current study, we undertook a comprehensive and unique approach to decouple lineage-related genes, combined with RPPA and targeted mutation analysis, and we identified immune and metabolic signatures that contributed to AML heterogeneity and impacted outcomes.

Our analysis identified a group of AML patients (group 2) who had significantly improved overall survival, event-free survival, and remission duration. This patient group was characterized by increased frequency of *GATA2* mutations and an inflammatory and immune phenotype indicated by enrichment for interferon-alpha and interferon-gamma, tumor necrosis factor-alpha, and interleukin-6/JAK/STAT3 signaling pathways. Interestingly, germline deficiencies in *GATA2* leads to myeloid malignancies with an immunodeficient phenotype (31). However, the exact mechanism by which *GATA2* mutations could confer a remodeled immunologic phenotype in AML remains unclear and warrants further investigation. Supported by previous studies demonstrating distinct immune cell activity among AML patients with different outcomes (32). Our findings suggested that the intrinsic inflammatory and immune microenvironment in AML was associated with better outcomes and responses to therapy. Recent work demonstrated the complex immunologic landscape of hematologic malignancies with a subset of AML patients having a distinctively high NK/T cell cytotoxic activity {Dufva:2020bg}. Further, recent work demonstrated that an immune-infiltrated signal was associated with improved outcomes in AML patients but not associated with ELN (33). However, in our results, which were validated in 2 independent cohorts, patients could be grouped by shared biologic characteristics independent of ELN classification or clinical variables such as age.

AML patients in group 2 also had significant downregulation of *HOX* genes, which corresponded with improved outcomes, consistent with previous studies (30, 34, 35). Inflammation and cytokine production in a canine model was associated with reduced *HOXA* gene expression (36), and restoring *HOX* gene expression may oppose inflammation (37) or hamper innate immunity by inhibiting granulopoiesis (38). These studies, although not conducted in a leukemia or cancer model, suggested that inflammation and *HOX* genes may be co-regulated, but the exact mechanism linking these two pathways is still unclear.

Our analysis also revealed 2 distinct patient groups (groups 1 and 3) that had similarly worse outcomes compared with group 2 but distinct underlying biology. Our orthogonal RPPA and genomic analysis coupled with transcriptomic profiling revealed that these 2 groups can be distinguished by increased metabolic activity and overexpression of mTOR and MCL1 proteins in group 3. However, only group 3 had *FLT3* enrichment, contrary to previous transcriptomic studies (17), demonstrating that multiple transcriptional clusters may harbor *FLT3* mutations. Therefore, our approach of decoupling the lineage-associated genes generated a better representation of the transcriptomic profile associated with *FLT3* mutations. This is also consistent with the proliferative phenotype conferred by *FLT3* mutations in AML (39). *FLT3* activates downstream mTOR signaling (40, 41), and this signaling is involved in metabolic reprogramming (42). Inhibiting mTOR can also lead to inhibition of MCL1, but the exact mechanism is not clear, although it is thought to involve AKT-dependent regulation of MCL1 (43, 44). mTOR inhibition also has antitumor activity in AML (45–47), and our data suggest that mTOR activation is associated with venetoclax resistance. The finding is of high importance because it suggests an alternative therapeutic target to overcome venetoclax resistance (26). Furthermore, mTOR inhibition could be a surrogate for inhibiting MCL1, especially given that direct MCL1 inhibitors have cardiac and gastrointestinal toxicities that have hampered their recent clinical development.

Our dataset comprised 81 samples from patients mostly treated with intensive chemotherapeutic regimens (80% with cytarabine-based regimens). Given the relatively small sample size, it is likely that we missed subtle transcriptomic and proteomic perturbations that might be biologically important. Furthermore, we used targeted sequencing of AML-associated genes to study DNA lesions in the cohort. Although this approach allowed us to study important AML-associated mutations in these patients, it precluded analysis of the full spectrum of mutations in these patients or the associated mutational processes, although most if not all of the myeloid mutations can be captured by this assay. Outcomes in patients with *FLT3* mutations (primarily group 3) would have been improved had FLT3 inhibitors been used. However, at the time of sample collection and AML diagnosis, none of the FLT3 inhibitors were approved or under investigation in a trial. Nevertheless, our study, which combined RPPA, genomic profiling, and transcriptomic profiling with extensive and long clinical follow-up data, provided a unique clinical dataset for further interrogation.

Our approach to decouple morphology from lineage-associated genes in AML revealed distinct groups of AML patients that share biologic pathways independent of ELN classification, antecedent hematologic disorders, or other clinical and molecular variables that are known to impact outcomes. We also used orthogonal RPPA analysis to differentiate patients with similarly worse outcomes in groups 1 and 3, revealing an mTOR-associated metabolic profile that can be potentially targeted. Our findings demonstrate that employing alternative classifications for AML patients can provide insight into AML heterogeneity.

## Data Availability Statement

The original contributions presented in the study are publicly available. This data can be found at: https://www.ncbi.nlm.nih.gov/geo/query/acc.cgi?acc=GSE165656.

## Ethics Statement

The studies involving human participants were reviewed and approved by IRB approval MD Anderson. The patients/participants provided their written informed consent to participate in this study.

## Author Contributions

HA, VM, and RW analyzed the data. YH and SL conducted the metabolic profiling. FW and JZ conducted DNA analysis. YQ and CH prepared samples. AQ analyzed RPPA. AQ and CB conducted sequencing. ND, MK, and AF contributed to clinical and integrative analysis. KC, LW, SK, and HA supervised the study. All authors contributed to the article and approved the submitted version.

## Funding

The work was funded in part by a T32 National Institutes of Health fellowship to HA. This project has been made possible in part by grant RP180248 to KC from Cancer Prevention & Research Institute of Texas and grant U01CA247760 to KC. RNA sequencing and Foundation Medicine assay sequencing were partially done *via* funding from Genentech to SK.

## Conflict of Interest

ND has received research funding from Daiichi-Sankyo, Bristol-Myers Squibb, Pfizer, Gilead, Sevier, Genentech, Astellas, Daiichi-Sankyo, Abbvie, Hanmi, Trovagene, FATE, Amgen, Novimmune, Glycomimetics, and ImmunoGen and has served in a consulting or advisory role for DaiichiSankyo, Bristol-Myers Squibb, Pfizer, Novartis, Celgene, AbbVie, Astellas, Genentech, Immunogen, Servier, Syndax, Trillium, Gilead, Amgen and Agios. MD and CB are employees of Genentech. MK reports grant support and consulting fees from AbbVie, Genentech, F. Hoffmann La-Roche, Stemline Therapeutics, Forty-Seven, consulting fees from Amgen and Kisoji, grant support from Eli Lilly, Cellectis, Calithera, Ablynx, Agios, Ascentage, AstraZeneca, Rafael Pharmaceutical, Sanofi, royalties and stock options from Reata Pharmaceutical Inc.

The authors declare that this study received funding from Genentech. The funder had the following involvement with the study: the funder was involved in the Foundation Medicine mutation calling.

The remaining authors declare that the research was conducted in the absence of any commercial or financial relationships that could be construed as a potential conflict of interest.

## Publisher’s Note

All claims expressed in this article are solely those of the authors and do not necessarily represent those of their affiliated organizations, or those of the publisher, the editors and the reviewers. Any product that may be evaluated in this article, or claim that may be made by its manufacturer, is not guaranteed or endorsed by the publisher.

## Supplementary Material

Supplementary Table 1 List of French American British (FAB)-associated genes and differentially expressed genes across all comparisons, in univariate and multivariable analysis, and between groups.

Supplementary Figure 1 Kaplan-Meier curves for **(A)** overall survival, **(B)** event-free survival, and **(C)** remission duration for all 81 patients in the cohort.

Supplementary Figure 2 **(A)** Clustered heatmap of top 1000 variably expressed genes in our cohort. Two patient groups were identified that showed strong association with French American British (FAB) status (Fisher p = 9.2e^-5^). **(B)** Clustering of samples performed as in **Supplementary Figure 1A** for various numbers of top variably expressed genes and their association with FAB status, inferred using the Fisher test. The analysis was also performed for clusters corresponding to **Figure 1A** (corrected_clusters). The barplot is the –log_10_ p values obtained from the Fisher test. **(C)** Transcriptome clustering analysis in The Cancer Genome Atlas (TCGA) acute myeloid leukemia cohort using the top 1000 variably expressed genes. Consistent with observations in our data **(A, B)**, identified clusters showed a strong association with FAB status (Fisher p = 2.24e-10).

Supplementary Figure 3 **(A)** Enrichment of cell type markers in genes associated with French American British classification. Strong enrichment was observed for genes associated with myeloid and monocytic lineage. **(B)** boxplot of expression of FLT3 between groups (ANOVA p = 0.025), Tuckey post-hoc test is used to compute q-values for pairwise comparisons. **(C)** Heatmap of mutations that are associated with FLT3 mutations (q < 0.1), identified using Fisher’s test followed by correction of p-value by FDR.

Supplementary Figure 4 Samples stratified on the basis of activity of *HOXA* or *HOXB* clusters and divided into high (above the fiftieth percentile) and low (below the fiftieth percentile) groups. Survival between these groups was compared in **(A)** the MD Anderson cohort (top: overall survival; bottom: event-free survival), **(B)** the Valk et al. cohort (top: overall survival; bottom: event-free survival), and **(C)** The Cancer Genome Atlas (TCGA) cohort (overall survival).

Supplementary Figure 5 **(A)** Proteins differentially expressed in group 2 relative to group 1. Upregulated proteins are shown in red (q < 0.1 and difference in mean > 0.149) and downregulated pathways in blue (q < 0.1 and difference in mean < -0.117). **(B)** Proteins differentially expressed in group 2 relative to group 3. Upregulated proteins are shown in red (q < 0.1 and difference in mean > 0.22) and downregulated pathways in blue (q < 0.1 and difference in mean < -0.24).
