# Supplementary material for: Decoupling Lineage-Associated Genes in Acute Myeloid Leukemia Reveals Inflammatory and Metabolic Signatures Associated With Outcomes
Source: Front Oncol. 2021 Aug 4;11:705627. doi: 10.3389/fonc.2021.705627 (PMC8372368; doi:10.3389/fonc.2021.705627)

Supplementary Figure 1

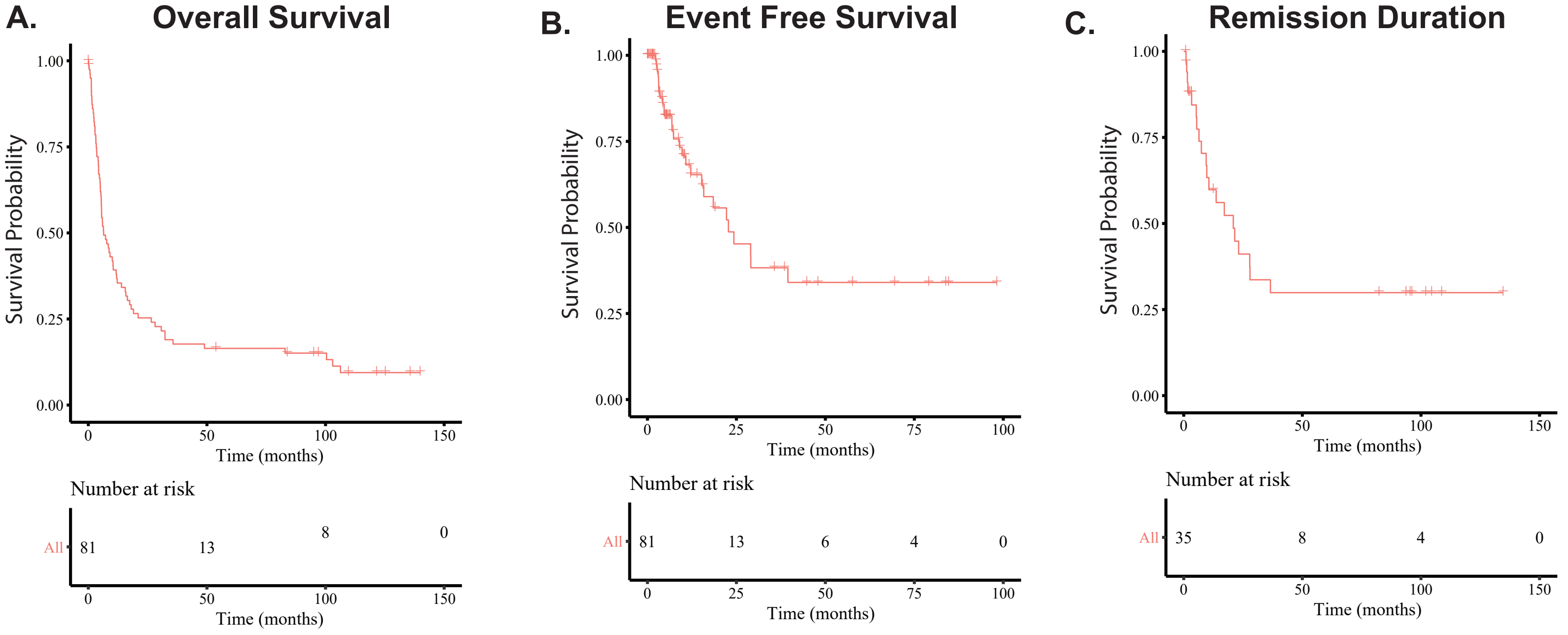

Supplementary Figure 2

A.

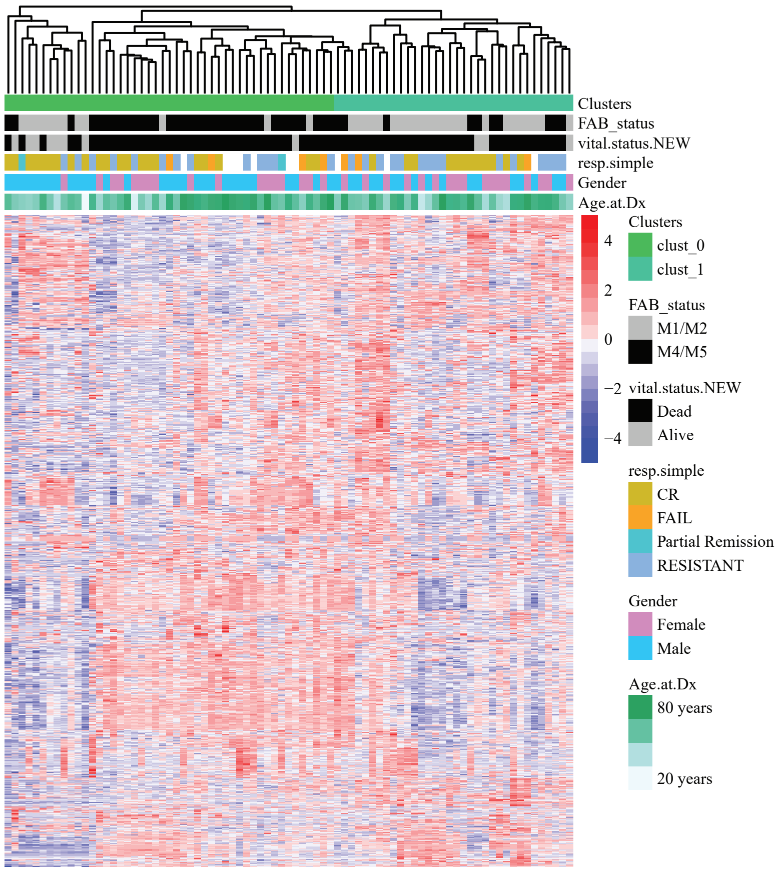

B.

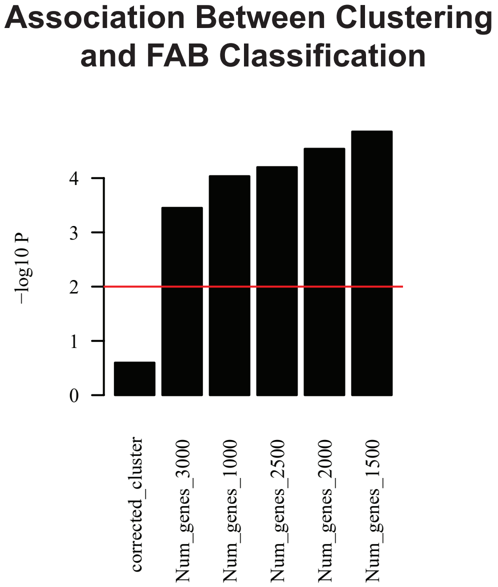

C.

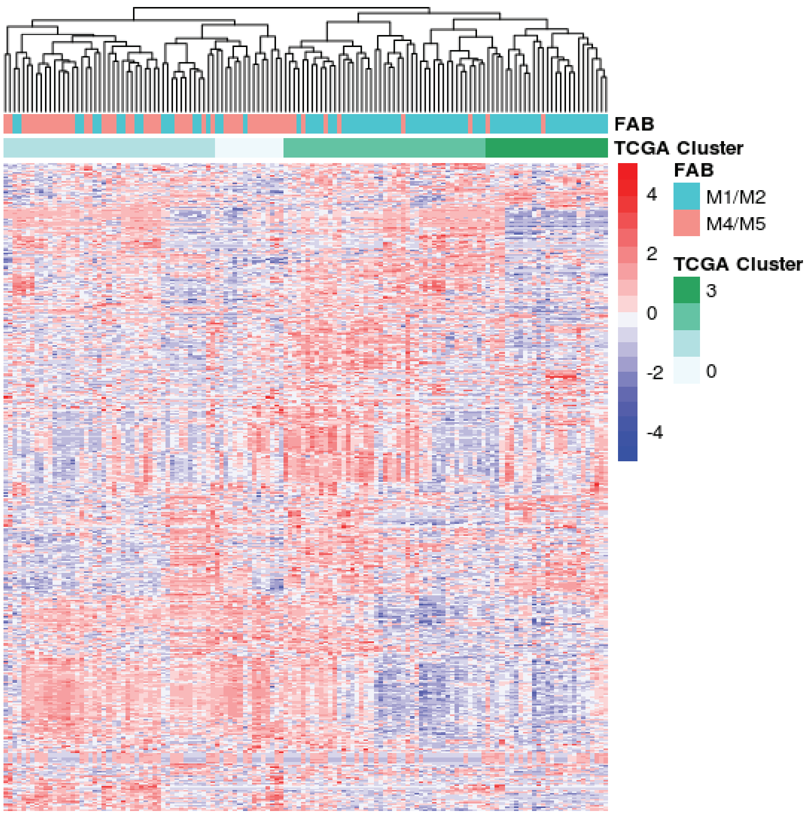

Supplementary Figure 3

A.

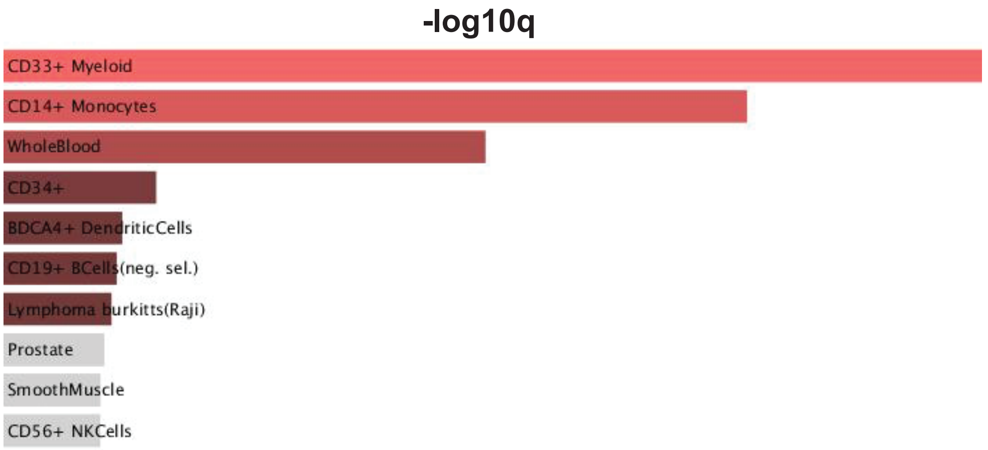

B.

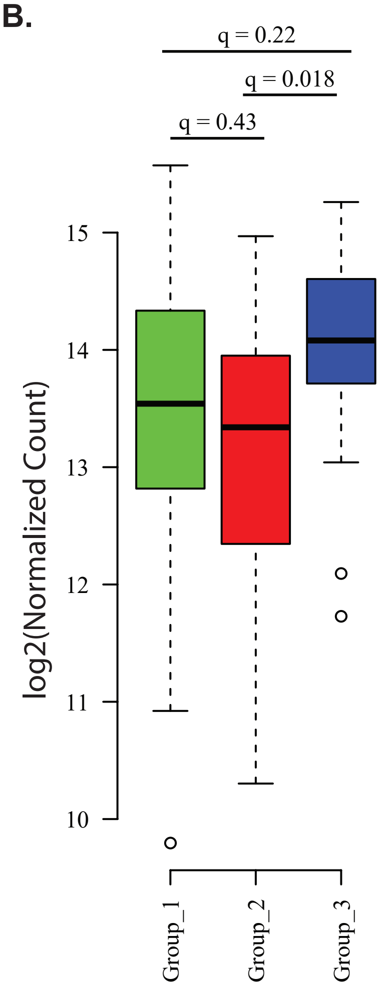

C.

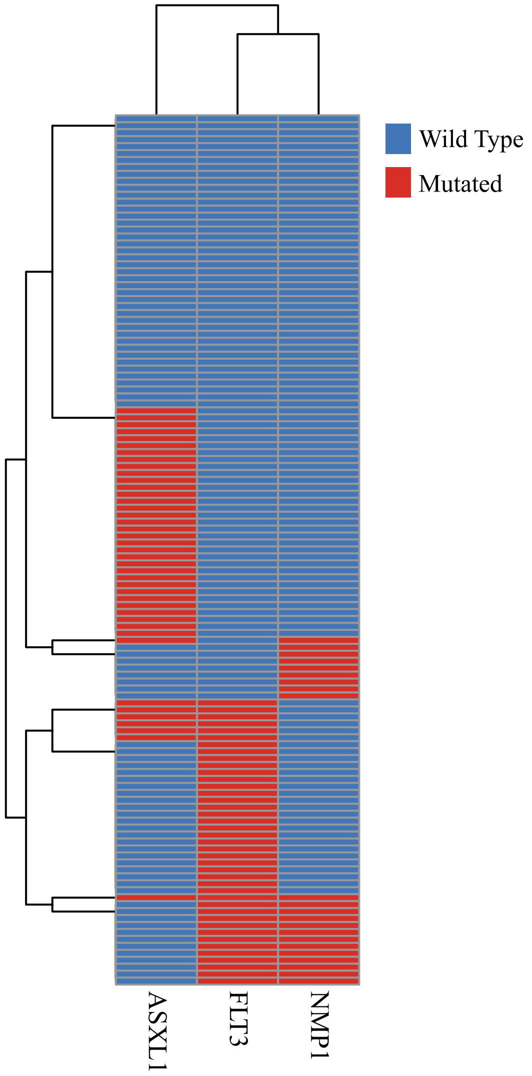

Supplementary Figure 4

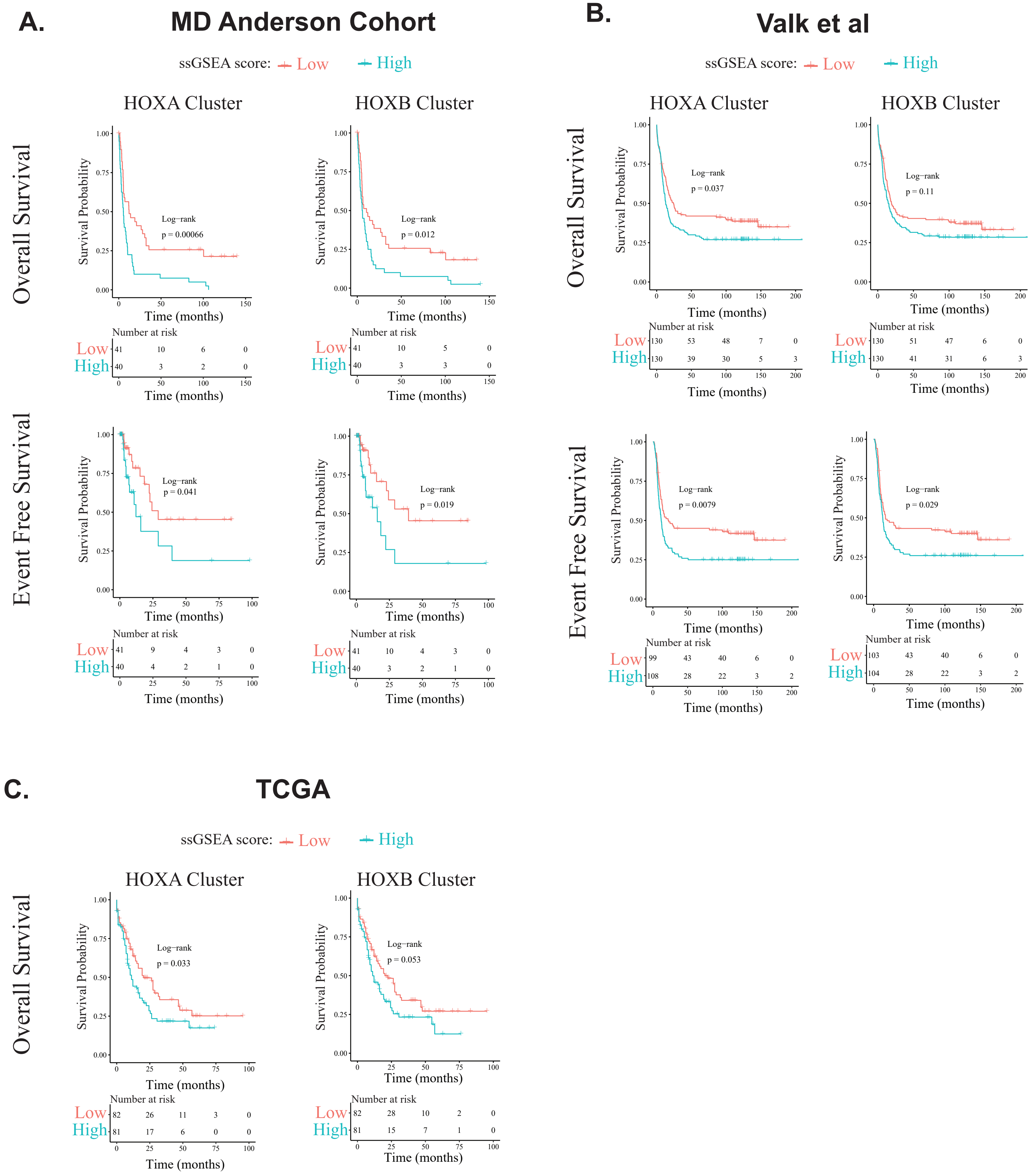

Supplementary Figure 5

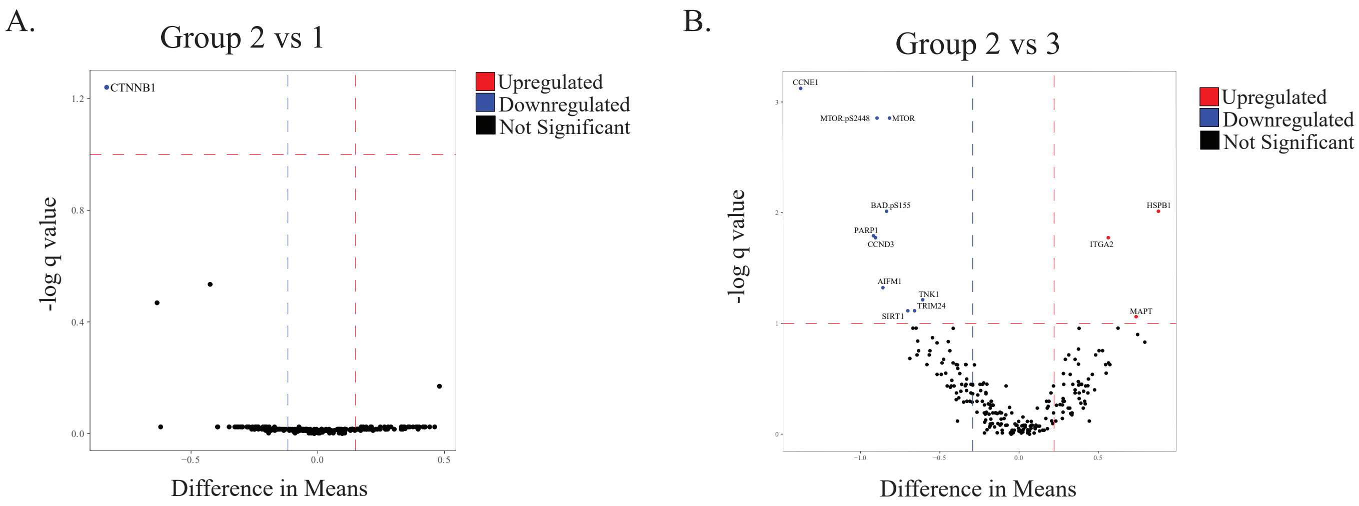

Supplement: Supplementary Figure 1 — Kaplan-Meier curves for (A) overall survival, (B) event-free survival, and (C) remission duration for all 81 patients in the cohort. [file DataSheet_1.pdf]
